# Employability Appraisal Scale (EAS): Development and Validation in a Spanish Sample

**DOI:** 10.3389/fpsyg.2018.01437

**Published:** 2018-08-14

**Authors:** Lucía I. Llinares-Insa, Pilar González-Navarro, Juan J. Zacarés-González, Ana I. Córdoba-Iñesta

**Affiliations:** ^1^Department of Social Psychology, University of Valencia, Valencia, Spain; ^2^Department of Social Psychology, IDOCAL, University of Valencia, Valencia, Spain; ^3^Department of Developmental and Educational Psychology, University of Valencia, Valencia, Spain

**Keywords:** employability, scale development, validation, psychometric properties, job inclusion, job loss

## Abstract

Employability is an important issue in the labor context. Currently, the European Union presents employability as the path to full employment and active citizenship, and a strategy to reduce unemployment and poverty. This study develops and validates an Employability Appraisal Scale. Specifically, we propose a multidimensional employability scale that analyzes both individual indicators and personal circumstances from the Bioecological Model of Employability. The Employability Appraisal Scale (EAS) assesses personal and social dimensions of employability. It was developed and tested using data from 489 people from a very heterogeneous sample (precarious workers, professionals, prisoners, long-term unemployed, socially excluded, etc.). Results provide evidence for the multi-dimensional structure and validity of the EAS. This scale is a valid and reliable instrument to measure employability, and it provides criteria for interpreting scores. Finally, we present theoretical and practical implications of the EAS for social and labor integration, job transition, and career development. Our findings have positive implications for identifying effectiveness indicators in training programs, and they contribute to designing intervention policies to increase employability.

## Introduction

Employability is a key issue in the labor context. In fact, current economic policies, new job and career models, and high unemployment rates highlight the role of employability (Hillage and Pollard, 1998; Van der Heijden, 2002; Bonfiglioli et al., 2006; ILO, 2017). The European Union presents employability as the path to full employment and the promotion of active citizenship (SEC, 2000), and as a strategy to reduce unemployment (ILO, 2017) and poverty (European Commission, 2010; ILO, 2016). Therefore, employability is an important issue that warrants further study (European Council, 2015; European Parliament, 2015).

Research that has analyzed the concept of employability has mainly concentrated on personal factors (Fugate et al., 2004), leaving out other factors such as the employee's circumstances. This represents an important gap because some job skills needed to access employment are dependent on cultural, economic, and labor conditions. In this context, the Bioecological Model of Employability (Llinares et al., 2016) provides a holistic view that focuses not only on the individual's responsibility for his/her career development, but also on contextual factors. In this regard, employability can be seen as a transversal meta-competence that develops through a process that connects the whole person with the acquisition and maintenance of employment. This meta-competence aids in the acquisition of other important competences (Winterton, 2009). Thus, according to the appraisal model by Lazarus and Folkman (1984), employability will affect cognitive, emotional, and behavioral aspects of the job search and job maintenance.

From this perspective, this meta-competence represents a transversal and essential standard for any profession, class, gender, etc. Acquiring knowledge about this employability meta-competence fills an important gap for several reasons. First, when the literature examines employability in specific samples (e.g., graduates, Gogoi, 2016; post-graduates, Rothwell et al., 2009; women, Roy and Mukherjee, 2013), this type of employability only refers to a particular sample or a particular job. In order to achieve a better measure of employability across different samples and contexts, we intend to assess some essential standards for employability (such as EAS). Second, as far as we know, employability scales do not usually use a theoretical framework (González-Romá et al., 2018). This paper presents a scale based on the Bioecological Model of Employment. Third, there is a lack of agreement about what specific factors are important in assessing employability (Tymon, 2013), or scales may assess only one domain of employability (e.g., De Witte, 2005; De Cuyper et al., 2008). However, the assessment of essential standards through the EAS provides information about cognitive, emotional, and behavioral appraisals of employability. Fourth, currently researchers and international organizations (e.g., International Labor Organization, United Nations, European Commission, …) are trying to reduce poverty and unemployment (e.g., European Commission, 2010) and provide guidelines for employment policies (European Council, 2015). Thus, they defend the importance of increasing employability. This requires a validated employability scale that identifies intervention domains. We propose the EAS as a way to fill this gap because it integrates standard individual characteristics and contextual factors that are essential for employability.

The main goal of this study is the development, validation, and evaluation of the psychometric properties of the employability appraisal scale. For this purpose, we used domains of the Bioecological Model of employability (personal and contextual factors) that are important in ensuring career opportunities.

Our study attempts to make a number of contributions. First, by using a heterogeneous sample, we offer a multi-domain scale that can be widely used. Thus, we extend previous scales and design a scale that can be used with any age group, social class, gender, ethnic group, or educational level. Second, the assessment of employability should include essential standards for labor integration. In this regard, we propose a scale that is useful for the assessment and individual promotion of employability in vulnerable groups (González Barriga, 2003; Llinares et al., 2011). Third, from a practical perspective, our scale allows human resources professionals to orient people's career development. Thus, it provides a guide for suggesting ways to foster employability and improve inclusion in the job market. Fourth, our measure is a useful and valid scale that includes 35 items; therefore, its application is fast and easy, regardless of training, culture, ethnicity, gender, age, social class, or job situation.

## Theoretical framework

### The bioecological model of employability

Employability is one of the key elements to analyze in recovering from the current economic crisis, enhancing the labor supply and the functioning of labor markets, and developing new career models (De Cuyper et al., 2011). In general, public discourse has had a superficial view of employability. In this regard, employability has experienced socio-historical swings, and it has a large number of meanings, even more so than other psychosocial constructs (e.g., identity) (Hillage and Pollard, 1998; Bonfiglioli et al., 2006). In addition, employability is usually understood as gaining and retaining employment and the idea that the person is responsible for his/her own career path (e.g., Van der Heijde and Van der Heijden, 2006), without conferring any responsibility to other social agents. This perspective is reflected in models that define employability as a product of individual differences, specific characteristics, and experiences that distinguish the employable person from the non-employable one (Fugate et al., 2004). For example according to Fugate et al. (2004), the concept of employability includes these person-centered dimensions: career identity, personal adaptability, and social and human capital. However, a critical perspective addresses the socio-historical nature of employability, and asserts that, by focusing on individual differences, the positions of power justify the exclusion of certain people and groups based on their particular characteristics (Lindsay and Serrano, 2009). Drawing on this critical perspective, we understand that employability would integrate individual and contextual variables, and we use the four components (proximal processes, biopsychological characteristics of a developing person, parameters of the ecological context, and the temporal dimension), proposed by Bronfenbrenner's Bioecological Model of Employability (Llinares et al., 2016), to develop a holistic definition of employability.

The bioecological perspective considers employability to be the result of personal and contextual factors, combining individual characteristics and proximal processes. With this model, we consider employability to be a personal meta-competence (first key element), but, at the same time, a social construction. The person is active and intentional. Just as in the process of anticipatory socialization, the person develops a meta-competence of employability that integrates other important competences (Winterton, 2009), that is, a set of behaviors, knowledge, thought processes, and/or attitudes at an elementary, basic, or high performance level (Warr and Conner, 1992).

The second key element of employability involves approaching it as a social construction. Employability must be considered within the context of a particular social system, and it is a product of social regulations and power relations among social groups. From this perspective, employability is a process that builds on the individual and group history of individuals and societies. The social context is a set of nested structures surrounding the person (Bronfenbrenner, 2005), which leads us to identify another issue in the emerging debates on employability, defining its components (Rothwell et al., 2008) or set of indicators (Llinares et al., 2016). These indicators allow us to recognize competences for training, learning, and assessment (e.g., Buchmann, 2002; Fugate et al., 2004; McQuaid and Lindsay, 2005).

Therefore, it is important to have an employability scale with a holistic perspective that integrates all the elements proposed by the Bioecological Model. The present research focuses on the validation of a new scale to capture employability as a meta-competence, using the Bioecological Model in the labor context.

### Scales and domains of employability

Currently, the scientific literature on employability demands valid measures this construct (e.g., Jonck and Minnaar, 2015), and Work Integration Social Enterprises (WISE) defend the need for training in employability (e.g., G20 Employment Working Group, 2016). There are a large number of existing batteries of questionnaires (e.g., Su and Zhang, 2015) and/or interviews (e.g., Harvey, 2001; Van den Hof, 2015). Some of them analyze employability by focusing on the perception of control over achieving employment (e.g., De Witte, 2005), whereas others focus exclusively on the person (e.g., Qenani et al., 2014) or enterprise (e.g., Rothwell and Arnold, 2007).

One of the most widely used scales (see Córdoba et al., 2013) is the Competence-based and multidimensional measure of employability by Van der Heijde and Van der Heijden (2006). This scale focuses on the most relevant competences in workers to improve their employability and benefit both the employee and the employer. It distinguishes among five dimensions of employability: occupational expertise, anticipation and optimization, personal flexibility, corporate sense, and balance. Moreover, the Self–perceived employability scale (Rothwell and Arnold, 2007; Rothwell et al., 2008, 2009) attempts to capture what employability really means to individuals within the context of their experiences, aspirations, and perception of their ability to compete in the external labor market. In addition, the Dispositional measure of employability (DME) (Fugate and Kinicki, 2008) measures dispositional employability. The authors define it as a constellation of individual differences that predispose individuals to proactive adaptability, specifically to work and careers, i.e., the individual's ability to adapt to changes in the labor market in order to stay employable.

Most of these scales assess individual characteristics, but not the social context. Moreover, from an economic point of view, these scales are designed to differentiate between employable and unemployable people (e.g., Salognon, 2005, 2007). Nevertheless, from the bioecological perspective of employability, a scale should assess a single meta-competence for everyone (temporary workers, qualified workers, unemployed people, women, young people, immigrants, etc.). In this regard, the indicators analyze the itineraries for the integration in social enterprises, and they highlight individual and social elements (McQuaid and Lindsay, 2005; Llinares et al., 2016). Individual elements refer to the worker and include personal attributes (honesty, disposition for work, intrinsic motivation, attitudes toward work); skills and competences (basic abilities, adaptability, work or social skills, such as teamwork or positive management of conflict); and health or geographic mobility and work flexibility. Social elements condition performance at work and include family responsibilities, the work culture, and access to resources such as transport, housing, social support, and conditioners related to the job market, macro-economic factors, or employment policies (Córdoba et al., 2013).

To the best of our knowledge, there are no statistically validated instruments that evaluate both elements of employability (e.g., Saad et al., 2013; Karli, 2016). Therefore, the goal of the present study is to develop and validate an employability appraisal scale that takes both dimensions into account.

## Study 1. qualitative study of the scale's development: item generation

The aim of the first study is to develop an appraisal scale to measure employability. We expect a multi-dimensional structure for this scale (Hypothesis 1), so that it can be applicable to different social groups (unemployed people, workers, women, immigrants, etc.). In this study, we describe the process of constructing the scale. Thus, in Study 1, we created a questionnaire, following the guidelines of Simms and Watson (2007).

### Method

Following Simms and Watson (2007), we began with a review of the literature on measures of employability. However, we did not find any measures that assess individual and contextual elements of employability. To select and develop appropriate items, we used two inclusion criteria: first, indicators used for groups at risk of social exclusion or vulnerable groups (Llinares et al., 2011); second, indicators that analyze the person's maturity from a bioecological and social perspective (Llinares et al., 2016). For the first criterion, employability indicators were used that had been applied and previously found to be relevant in groups of workers at risk of social exclusion. Specifically, indicators were obtained from the employability assessment instruments used in Work Integration Social Enterprises (WISE), thanks to a collaboration agreement with the Federation of Associations of Integration Enterprises (FAEDEI, Spanish acronym). WISE are companies set up with the main purpose of providing jobs for people who, for several reasons, have not been able to find employment in an ordinary firm, even though they have the capacity to be productive. The purpose of WISE, therefore, is the training and social and labor integration of people in situations of social exclusion, by serving as a transition context toward ordinary employment. In this process, several individual and contextual indicators are collected about the progress of these workers in their training itinerary, clearly considered “employability indicators.” The second criterion involves obtaining indicators that, fulfilling the previous criterion, correspond to the second facet of the bioecological model concerning the biopsychological characteristics, which affect the level of employability of each individual. Three types of personal attributes are considered relevant to the analysis of employability (Bronfenbrenner and Morris, 2006; Llinares et al., 2016): willingness (to develop themselves and to adapt to changes), resources (human and social capital), and demands (personal appearance and interpersonal behavior interacting with employers and managers). By combining the two criteria, thirteen significant employability indicators were finally chosen: perseverance, academic qualifications, professional qualifications, learning to learn, time management, task management, initiative, social skills, autonomy, will and willingness to work, specific professional skills, personal care, and work experience.

In this stage, first, we chose items from questionnaires or factors from scales used to measure each indicator. This study used an instrument consisting of 58 items that measure thirteen indicators: perseverance (e.g., “I persevere when I have to perform a long and difficult task”), stress, and frustration tolerance (e.g., “I get angry easily”), academic and professional qualifications (e.g., “My training is insufficient to work as a professional”), learning to learn (e.g., “I view changes as an opportunity to learn, and not as a difficulty”), time (e.g., “I have a tendency to leave things until the last minute”) and task management (e.g., “I can design a good plan of action when I have to do something important related to my studies or my work”), initiative (e.g., “I consider myself a person with initiative for beginning tasks, making decisions, or solving problems”), social skills (e.g., “I can't find a job because I lack the ability to express myself and relate to other people”), autonomy (e.g., “I have confidence in my own opinions, even if they are different from other people's”), will and willingness to work (e.g., “I am a practical person. I know what I have to do and I do it”), professional skills (e.g., “I can't find a job because I don't keep up with my profession and I'm not competent”), personal care (e.g., “I have a bad appearance, and I think that is why I can't find a job”), and work experience (e.g., “I do not have enough experience to be hired”). Items were checked and adapted from the Mature Person Traits Questionnaire (Zacarés and Serra, 2000, in Nuñez, 2015), the Scale of Time Usage (Feather and Bond, 1983), the Questionnaire on expectations of socio-professional insertion (Figuera, 1994), the Questionnaire on the attribution of employment (Villar, 1991), and Stress Management from the Emotional Quotient Inventory (Bar-On and Parker, 2000).

Next, to verify the content validity and applicability of the first version, two processes were carried out. In the first one, we asked nine experts to analyze the adequacy of the items for the theoretical dimension on a 5-point Likert scale. This multidisciplinary team of judges consisted of Education professionals, Education and developmental psychologists, Sociologists, Social psychologists, and Social workers. Based on their observations, we elaborated the second version of the measurement instrument using inter-rater agreement (*k* = 0.89) to eliminate items that caused confusion. After that, we performed a pilot test in a small convenience sample (*n* = 162). This sample was composed of long-term unemployed people, active workers with low and medium qualifications, and volunteer university students. We asked them to fill out the questionnaire and add their observations, criticisms, and suggestions. Their answers allowed us to assess the effectiveness and relevance of the questionnaire, the difficulty of understanding some words, possible ambiguities, missing information, or adequate length.

Next, we elaborated the third version of the measurement instrument, which was assessed by the multidisciplinary inter-rater team as well as accompanying workers and WISE managers (*k* = 0.84). Finally, we prepared an introduction with the instructions for filling out the questionnaire, guaranteeing confidentiality, anonymity, and voluntary participation, and acknowledging their collaboration in the research. Thus, we obtained the final version of the scale: “Employability Appraisal Scale (EAS).”

### Results

The EAS measures the basic individual and social elements of employability; specifically, it collects the main cultural elements related to anticipatory socialization and the level of maturity. Therefore, the score does not show the probability of getting a job, but rather the adequate level of the meta-competence to get and retain a job.

The Spanish final 35-item version of the scale (Table 1) was measured using a 5-point Likert scale, and it has seven classification variables (age, gender, studies, complementary studies, nationality, illness/disability/dependence, and legal processes) and seven variables focused on social elements (civil status, number of children, people living at home, dependent people at home, time flexibility and availability, and work experience).

**Table 1:** Factor structure, Items on the EAS (Spanish and their English translation), and EFA four-factor model.

**Spanish items on the EAS**	**English items on the EAS**	**Loadings**
**FACTOR 1. EMPLOYMENT PROTECTIVE BEHAVIORS**		
1. Consigo lo que me propongo hacer	1. I achieve what I set out to do	0.69
2. Confío en mis opiniones incluso si son diferentes a las de los demás	2. I have confidence in my own opinions, even if they are different from other people's	0.44
3. Me pongo a trabajar cuando decido qué quiero hacer	3. I get to work when I decide what I want to do	0.50
9. Soy capaz de organizar mi trabajo cuando tengo que hacer algo importante para mis estudios o mi trabajo	9. I can design a good plan of action when I have to do something important related to my studies or my work	0.54
12. Me implico mucho en lo que hago y me gustan las tareas que comienzo a hacer	12. I get involved in what I do, and I am enthusiastic about the tasks I undertake	0.67
13. Para mí, es más importante sentirme bien conmigo mismo que recibir la aprobación de los demás	13. For me, it is more important to feel good about myself than to receive the approval of others	0.68
15. Soy eficaz en mi trabajo.	15. I consider myself effective in my work	0.79
20. Soy una persona responsable de todo lo que hago y decido	20. I am responsible for my actions and decisions	0.65
25. Soy una persona práctica, sé qué tengo que hacer y lo hago	25. I am a practical person. I know what I have to do and I do it	0.56
27. Organizo mi tiempo y lo aprovecho al máximo	27. I can organize my time to make the most of it	0.49
29. Soy persistente y constante. Termino lo que empiezo	29. I am persistent and tenacious. I finish what I start	0.59
34. Soy una persona con iniciativa para comenzar las tareas, tomar decisiones rápidas o buscar soluciones a problemas.	34. I consider myself a person with initiative for beginning tasks, making decisions, or solving problems	0.67
	**FACTOR 2. EMPLOYMENT RISK**	
4. Me aburro haciendo las actividades de la vida diaria	4. I get bored with doing daily activities	0.52
5. Suelo cambiar muchas veces de actividad durante el día sin un fin concreto	5. I have a tendency to change activities during the day without a specific reason	0.52
7. No consigo ser constante cuando debo realizar una tarea larga y difícil	7. I do not persevere when I have to perform a long and difficult task	0.65
10. No tengo suficiente formación para poder trabajar	10. My training is insufficient to work as a professional	0.39
11. No tengo la experiencia suficiente para que me contraten	11. I do not have enough experience to be hired	0.41
14. Hay otros profesionales mejor preparados que yo para trabajar	14. There are other professionals better prepared to work than I am	0.36
16. Suelo dejar las cosas para el último momento	16. I have a tendency to leave things until the last minute	0.61
18. Cuando tengo que hacer algo me cuesta mucho ponerme en marcha	18. When I have to do an activity I take a long time to get going	0.72
19. Suelo tener problemas para organizarme las tareas que debo hacer	19. I have problems organizing the things I have to do	0.69
23. Creo que no puedo hacer todas las actividades que deben hacerse todos los días	23. I have the impression that I cannot do the activities that need to be done every day	0.54
	**FACTOR 3 JOB-SEEKING BEHAVIOR (REVERSE SCORING)**	
6. Tengo mala presencia y creo que esto hace que no encuentre trabajo	6. I have a bad appearance and I think that is why I can't find a job	0.76
24. No encuentro trabajo porque no sé cómo buscar trabajo	24. I can't find a job because I don't know how to look for one	0.81
26. No encuentro trabajo porque tengo problemas para expresar lo que pienso y relacionarme con otras personas	26. I can't find a job because I lack the ability to express myself and relate to other people	0.78
28. No encuentro trabajo porque me falta confianza en mí mismo.	28. I can't find a job because I lack self-confidence	0.76
30. No encuentro trabajo porque debo ser más constante cuando lo busco y no desanimarme	30. I can't find a job because I have to be more persistent when I search for employment and not get discouraged	0.71
31. No consigo trabajo porque no estoy al día de mi profesión y no soy competente	31. I can't find a job because I don't keep up with my profession and I'm not competent	0.27
	**FACTOR 4. SELF-CONTROL (REVERSE SCORING)**	
17. Me resulta difícil controlar mi ira, rabia, malestar.	17. I find it difficult to control my anger	0.60
21. Hay cosas que me enfadan y molestan mucho	21. Some things annoy me a lot	0.83
32. Me enfado mucho con facilidad	32. I get angry easily	0.52
33. Tengo mal genio	33. I have a bad temper	0.86
	**FACTOR 5. SELF-LEARNING**	
8. Cuando necesito saber algo en mi trabajo, suelo preguntar o pedir que me enseñen	8. When I need to know something at work I usually ask or ask to be taught	0.78
22. Me gusta aprender cosas nuevas sobre mi trabajo incluso si se trata de pequeños detalles	22. I like to learn new things about my work even if it's about small details	0.80
35. Yo veo los cambios como una oportunidad para aprender y no como una dificultad	35. I view changes as an opportunity to learn, and not as a difficulty	0.35

## Study 2: quantitative study: validation of the EAS and psychometric properties

In Study 2, we conducted an Exploratory Factorial Analysis (EFA) to analyze the dimensions of the questionnaire (sample 1). Subsequently, a Confirmatory Factor Analysis (CFA) was carried out, and evidence for validity and psychometric properties were evaluated (sample 2).

### Methods

#### Sample and data collection

Data were collected through a self-administered questionnaire. Participants in sample 1 were 237 Spanish unemployed people. The gender distribution of this sample was 78.5% female and 21.5% male, with a mean age of 38.74 years (*SD* = 11.01), ranging from 18 to 67 years. In terms of their level of education, the majority had primary studies (27.5%), followed by university degrees (16.5%).

The participants in sample 2 were 252 Spanish workers in different industrial, commercial, and service organizations, unemployed people, and graduates. More women (64.1%) than men (39.5%) participated, with a mean age of 29.84 years (*SD* = 11.47), ranging from 18 to 68 years. In terms of their level of education, the majority had a university degree (62.9%), followed by elementary school (17%), vocational training (8.9%), and Bachelor's degrees (8.9%). Two samples were convenience samples with an adequate sample size (Lloret et al., 2014).

The questionnaires were filled out and gathered in the workplace, the Valencian Service of Formation and Occupation (“SERVEF”), and Social Services Offices. Participants received instructions and information about the procedure for filling out the questionnaire. Moreover, the presence of the researcher was helpful, as any doubts about the questions were resolved and clarified. The researchers emphasized that the data provided by the participants were anonymous, and that there were no right or wrong answers (Podsakoff et al., 2003). Missing data on conflictive items represented about 0.3%, and these participants were excluded from the analyses.

This study was carried out in accordance with the ethical guidelines of the American Psychological Association and the Declaration of Helsinki, and it received approval from the Ethics Committee of the University of Valencia (H1445854612881). Participation was completely voluntary, the answers were anonymous, the consent obtained from the participants was both informed and written, and the sample did not receive any monetary compensation.

#### Measures

Employability was measured with the Employability Appraisal Scale (EAS), with 35 items, reported in Study 1 (see Table 1).

Agreeableness was assessed with a 12-item factor, based on the Spanish version of the NEO-PI-R (Aluja et al., 2004). The answer format is a 5-point Likert-type scale (ranging from “Strongly disagree”-1 to “Strongly agree”-5) (“When I have been offended, I try to forgive and forget”). Cronbach's Alpha was 0.7.

Conscientiousness was assessed with a 12-item factor, based on the Spanish version of the NEO-PI-R (Aluja et al., 2004). A sample item is “I have clear objectives, and I strive to achieve them in an orderly way,” and items were scored on a 5-point Likert scale (from 1-Strongly disagree to 5-Strongly agree).

Problem Solving was assessed with one factor from the Spanish version of the Conflict Management Strategies in the Workplace scale (De Dreu et al., 2001). This scale consists of 5 items (e.g., “I accept that my goals and interests and those of the other party can be achieved"). The items are measured using 6-point scales, ranging from 1 (never) to 6 (often). Cronbach's Alpha was 0.74.

#### Data analysis

Statistical analyses were conducted using SPSS 22 and EQS 6.1 software.

First, Barlett's test and the Kaiser-Meyer-Olkin (KMO) were used to examine whether the data were appropriate for an Exploratory Factor Analysis (EFA) in sample 1 (Bartlett, 1954). Higher values indicate a stronger correlation between the items, which means that EFA is appropriate (Kaiser, 1974). When the KMO value is ≥0.60, EFA can be used (Tabachnick and Fidell, 2007). EFA with Unweighted Least Squares (ULS) and Kaiser criterion and Oblique rotation (direct oblimin) were used to identify meaningful components (Lloret et al., 2014).

Moreover, the internal consistency of a group of items was assessed by Cronbach's alpha (Cronbach, 1951) and Composite Reliability (CR). Both should be ≥0.6 to indicate good reliability (Fornell and Bookstein, 1982).

Second, in order to determine the factorial structure of the EAS in sample 2, as a preliminary step, the polychoric correlation matrixes among the items were obtained. The model generation phase began by fitting the initial model to the data (Jöreskog and Sörbom, 2006). Due to the ordinal nature of the item-level data (Likert scale), robust maximum likelihood was used for parameter estimation. Model fit was evaluated using absolute (Jöreskog and Sörbom, 2006) and relative indices (Marsh et al., 1996): (a) χ2 statistic with Satorra –Bentler correction and χ2/df<2 (Kline, 1998); (b) the Comparative Fit Index (CFI) and the Normed Fit Index (NFI) with a cut-off criteria of ≥0.90 (Hu and Bentler, 1999); and (c) the Root Mean Square Error of Approximation (RMSEA), with values ≤0.08 indicating good fit (Hair et al., 2006).

Two confirmatory factor analyses (CFA) were carried out. The structure derived from the theoretical considerations and the EFA was used as the baseline model to be estimated with confirmatory techniques (CFA). The second CFA was conducted to test a single-factor structure, and the results were compared to find the most parsimonious model.

Third, after the dimensionality of the questionnaire had been clarified, we evaluated the psychometric proprieties (reliability and criterion validity). The average variance extracted (AVE) was calculated (Fornell and Larcker, 1981) to evaluate discriminant validity. To explore concurrent and external validity, it is difficult to find scales related to employability. We decided to perform correlations with some transversal job demands. Job demands refer to factors of a job that require continuous psychological effort or skills (Bakker and Demerouti, 2007). Three job demands and skills in workplaces have received particular emphasis in European society: solving problems, communication skills (agreeableness), and Conscientiousness (Leroux and Lafleur, 1995; Mousawa and Elyas, 2016).

Fourth, scores on each subscale and on the entire questionnaire were calculated, and we proceeded to construct the norms for interpreting these scores. For this purpose, we determined whether the scores on each scale followed a normal distribution by performing the Kolmogorov-Smirnov test. If this was the case, we obtained the norms by transforming the direct scores into standard scores, with the advantages of the properties of normal distribution; if not, we obtained the percentile norms.

### Results

Data were analyzed with the SPSS 22 program. We calculated the descriptive statistics (means and standard deviations) for the items in sample 1 and 2. Skewness and kurtosis values did not lie between −1 and 1; there was evidence of deviation from the normal distribution. The total item-scale and subscale correlation values were, in general, adequate.

EFA was carried out to examine the dimensionality of the scale for sample 1. The Kaiser-Meyer-Olkin (KMO) value was 0.79 and indicated that the sample data were suitable for factor analysis (Hair et al., 2006). Bartlett's Test (*p* < 0.001) showed that the correlation coefficients were not all zero. This confirmed the suitability of the data for factor analysis (Kaiser, 1960). The EFA performed with the 35 items showed a consistent internal structure (alpha factors ≥0.80), and explained variance was 41.9% in all the factors. Five factors were obtained with eigenvalues >1, following Kaiser's criterion. Table 1 presents the rotated factor loadings that exceeded 0.30 for the five-factor model. However one item (item 31) was < 0.30. Methodological arguments were not convincing enough to support its inclusion in the factor; however, it seemed justified to include the item in the factor on the basis of theoretical reasoning. Average extracted communalities were ≥0.5, which indicates that there was good fit with the factor solution. All the items were maintained because their factor loadings were ≥ 0.40. Therefore, as a final result of this first analysis, the initial 35-item scale was maintained and used to perform the CFA on sample 2.

Sample 2 is adequate because the value of KMO is greater than 0.5 (0.79). The Bartlett test was significant for sample 2 (χ^2^ = 3996.06; *p* < 0.001). Descriptive analysis was also performed on the items. The skewness and kurtosis values showed evidence of deviation from the normal distribution in sample 2. Therefore, the decision was made to use the robust Maximum Likelihood estimation method. The 35-item test was further subjected to CFA using EQS to validate the proposed item structure that emerged from the exploratory phase (Hinkin, 1998).

For the initial model (one-factor), the ratio of X^2^/df was 2.91, which was below the accepted cut-off value of <5.00. This model presented an adequate fit to the data. Next, an inter-correlated five-factor model was specified (see Table 1), agreeing with the results of the EFA study. This model also presented a satisfactory fit (see Table 2). Additionally, it presented a reasonable RMSEA (values of 0.03 indicate reasonable fit; Jöreskog and Sörbom, 2006) and satisfactory NFI (0.9) and CFI (0.9) (Hu and Bentler, 1999). Moreover, the chi-squared test to compare the two models (one/five-factor model) was statistically significant (Δχ^2^ = 946.06, Δdf = 10 χ, ρ < 0.01), and so we decided to keep the five-factor model. Furthermore, the significant reduction in the chi-square of one model compared to the other also suggested a better fit to the data (Tabachnick and Fidell, 2007). This last analysis ended the model generation phase. Next, we estimated the Average Variance Extracted (AVE); only two factors of the employment scale had AVE below 0.5. When considering Discriminant Validity, correlations (highlighted) did not exceed the AVEs of either of the Latent Constructs (Hair et al., 2006).

**Table 2:** Goodness of fit indices for confirmatory analysis of the three competing models.

**Scales**	**χ^2^**	**d.f**.	**χ^2^/d.f**.	**IFI**	**NNFI**	**CFI**	**RMSEA**
One-factor model	1647.52	560	2.91				0.08
Five-factor model	700.94	550	1.27	0.90	0.90	0.90	0.03

*χ^2^, chi-square; df, degrees of freedom; IFI, Incremental Fit Index; NNFI, Non-Normed Fit Index; CFI, Comparative Fit Index; RMSEA, Root Mean Square Error of Approximation*.

Concurrent validity of the EAS was examined through its relationships with Problem Solving, cooperation, agreeableness, and conscientiousness. To illustrate the relationships between the measures, Table 3 shows the descriptive statistics, with the means, standard deviations, and correlations for all the variables. In general, correlations were moderate (≤0.40); these correlations were a guarantee of adequate validity. The correlation among the five factors were lower.

**Table 3:** Means, Standard Deviations, and Correlations between variable study.

**Scale**	**Mean**	**SD**	**Factor 1**	**Factor 2**	**Factor 3**	**Factor 4**	**Problem solving**	**Conscientiousness**	**Agreeableness**
Factor 1. Employment protective behaviors	4.05	0.38					0.00	0.33^**^	0.04
Factor 2. Employment risk	3.73	0.75	0.30^*^				0.01	−0.43^**^	−0.20^**^
Factor 3. Job-seeking behavior	2.34	1.15	0.25^*^	0.26^*^			0.13^**^	0.03	−0.10^*^
Factor 4. Self-control	3.38	0.47	−0.08^*^	−0.11*	0.15*		0.00	0.19^*^	0.27^**^
Factor 5. Self-learning	4.09	0.73	0.03^*^	0.01^*^	0.22^*^	0.04^*^	0.2^**^	0.41^**^	0.17^**^

* p < 0.05;

** *p < 0.01; SD, Standard Deviation*.

Reliability analysis (internal consistency) was performed. Table 4 shows Cronbach's Alpha for each subscale. These values were above the recommended value (Nunnally, 1978). Moreover, the internal consistency of the five subscales was also assessed using the composite reliability (CR) by Bagozzi and Yi (1988) (Table 4). These findings indicated that the scale is a reliable and valid instrument for measuring employability.

**Table 4:** Cronbach's Alpha value and CR of the Subscales and correlations.

**Scale**	**Alpha**	**CR**
Factor 1. Employment protective behaviors	0.87	0.88
Factor 2. Employment risk	0.81	0.84
Factor 3. Job-seeking behavior	0.69	0.88
Factor 4. Self-control	0.79	0.80
Factor 5. Self-learning	0.67	0.72

*CR, composite reliability*.

Finally, we show the interpretation of the scores. We tested whether the raw-score distribution for all the scales approached normal distribution. The Kolmogorov-Smirnov test results were significant at *p* < 0.05. Therefore, the data were not normally distributed. The norms presented the transformation of the raw scores into their corresponding percentiles (Table 5). Percentiles provided a simple and adequate interpretation of these raw scores. To establish the statistical norms, based on quartile, we decided to use three categories: lower (25th percentile), average (50th percentile), and high (75th percentile) quartiles.

**Table 5:** Percentiles of the EAS subscales and challenge/threat for total sample.

**Percentile**	**F1**	**F2**	**F3**	**F4**	**F5**
10	3.20	1.7	1.16	2.75	3
20	3.41	2	1.50	3.00	3.33
25	3.5	2.2	1.66	3.25	3.66
30	3.58	2.3	1.67	3.3	3.7
40	3.80	2.4	2.00	3.50	4.00
50	4.00	2.6	2.16	3.75	4.33
60	4.16	2.78	2.50	3.8	4.33
70	4.25	2.9	3.00	4	4.66
75	4.33	3	3.16	4.00	4.7
80	4.50	3.1	3.50	4.25	4.95
90	4.83	3.5	4.16	4.75	5

## General discussion

The aim of this study was to develop and validate an employability inventory and evaluate it as a transversal meta-competence for everyone. The first study provides a scale to measure employability, and the second study shows the validation evidence and psychometric properties. We initially established the factor structure with EFA and replicated it with confirmatory factor analysis. Both analyses provided a good fit. Moreover, the results showed empirical support for the validity of the EAS in any group (e.g., unemployed people, students, women, immigrants, etc.). Finally, we proposed the statistical norms for the interpretation of the score. These results have a number of theoretical and practical implications that we discuss in the following sections.

### Theoretical implications

Employability is a relevant meta-competence, and its evaluation is important for unemployed persons and for career development. Our study supports the existence of five general factors of employability (employment protective factors, employment risk, job-seeking behavior, self-control, and self-learning), rather than a one-dimensional structure (e.g., De Cuyper et al., 2008). A number of empirical studies suggest the multidimensionality of employability (e.g., Campbell, 2003).

In addition, in order to achieve a more complete understanding of the key dimensions of employability, the results obtained support a multifactorial conception of employability based on the Bioecological Model (Llinares et al., 2016). There are five major inter-related factors, but with their own identity, and they include all the domains of employability highlighted by the EAS: protective behaviors, elements of risk for the maintenance and/or attainment of employment, self-learning, self-control, and job search. Although previous research proposed its multidimensionality with specific personal factors (e.g., González-Romá et al., 2018), our results also provide empirical evidence about social elements to improve employability. In doing so, our study adds new knowledge to the literature on employability by presenting a model that includes it as a set of both personal resources (“employability capital,” Peeters et al., 2017) and contextual factors (“gender gap,” Llinares et al., 2018) at the same point in time.

Finally, our study extends previous research by showing that employability is a multidimensional meta-competence that can be evaluated and taught regardless of specific human characteristics. Previous literature on employability has focused on groups of university students and more qualified workers (Van der Heijde and Van der Heijden, 2006; Rothwell and Arnold, 2007; Fugate and Kinicki, 2008). As far as we know, no studies have analyzed this model in vulnerable socio-personal profiles. Longitudinal future studies should test to what extent the five factors identified in these results are significant for the acquisition and maintenance of a job.

### Practical implications

Our findings show some practical implications. First, the EAS could provide a way to build a Knowledge Society, reduce poverty and unemployment, and guide employment and educational policies (ILO, 2017). Second, the introduction of this new employability measure is necessary because it implies some improvements in the processes of evaluation, guidance, and intervention in job transitions and career development. The EAS is an adequate instrument to measure employability because it presents validity, reliability, good psychometric proprieties, and criteria for interpreting scores. Therefore, social agents or human resource services can use it to diagnose employability and its improvement. Third, taking into account that the EAS helps to identify domains of employability, counselors could advise people (e.g., graduates, unemployed people, etc.) about programs and strategies to develop specific domains. Thus, individuals would also benefit because they would know how to increase their employability and develop their careers.

Finally, one of the most widespread demands in the entire field of EAS is the need to avoid discriminating (positively or negatively) against the vulnerable groups they serve and eradicate social welfare actions. In this regard, social agents need an employability scale that can be used to offer a career plan instead of a job: a plan that includes socialization, training courses, and anything that might facilitate the person's job search (Santos et al., 2004; ILO, 2015). The EAS is validated, short, easy to administer to any group (e.g., active workers, the unemployed, ex-drug addicts, university students, long-term unemployed, immigrants,…). Therefore, it is an appropriate instrument for managing human resources and improving job security (Ducci, 1998; Fugate et al., 2004).

### Limitations and strengths

Our study has a number of limitations that must be kept in mind. First, all the data were collected from the same source (self-ratings of participants), which might involve common method bias due to the fact that the respondent providing the measure of the predictor and criterion variables is the same person (Podsakoff et al., 2003). In order to minimize this concern, we included different types of scales in the questionnaire, and the scales were alternated with related scales. We also aimed to prevent socially desirable answers by framing participation as voluntary and confidential. Future studies could limit these biases by using contextual measures, such as external agents (e.g., support technicians from WISE, human resources personnel, etc.), to extract a complete employability score for the person.

Second, although the heterogeneity of the sample allows us to capture the variability of the construct (adults with very different training levels, different nationalities, etc.), it can also affect low AVE scores and correlations. Further research should use multi-group analysis and factorial invariance.

These limitations notwithstanding, our study also shows several strengths. First, the analysis suggests that the scales are reliable and valid enough to continue to be easily applied in future research. Second, by testing a representative sample of participants with different work and educational status, we were able to offer a transversal measure that can be used to compare different groups. In addition, our multi-dimensional approach to employability allowed us to capture the fundamental individual and social dimensions.

## Conclusion

This paper develops and validates the EAS, and demonstrates its the good psychometric properties, based on the Bioecological Model of Employability. The EAS has 35 items that appraise employability, organized in five dimensions: protective employment behaviors, employment risk, job-seeking behavior, self-control, and self-learning. Our findings can be used by researchers, human resource services, and social workers to examine the employability of workers/unemployed people and design intervention programs (Haasler, 2013; European Commission, 2016).

## Author contributions

LL-I reviewed the literature, prepared the introduction, analysis, and results, and wrote the discussion, taking into account the different references used in the paper. PG-N is the corresponding author. She reviewed the literature, prepared the introduction, analysis, and results, and wrote the discussion, taking into account the different references used in the paper. JZ-G reviewed the literature and selected the information, and he revised the discussion and the final English version. AC-I reviewed the literature and extracted the information to revise the discussion and the final English version.

### Conflict of interest statement

The authors declare that the research was conducted in the absence of any commercial or financial relationships that could be construed as a potential conflict of interest.
